# Artificial intelligence ethics in precision oncology: balancing advancements in technology with patient privacy and autonomy

**DOI:** 10.37349/etat.2023.00160

**Published:** 2023-08-31

**Authors:** Bahareh Farasati Far

**Affiliations:** University of Campania “L. Vanvitelli”, Italy; Department of Chemistry, Iran University of Science and Technology, Tehran 16846-13114, Iran

**Keywords:** Artificial intelligence, precision oncology, ethics, patient privacy, patient autonomy, machine learning, personalized medicine, data protection

## Abstract

Precision oncology is a rapidly evolving field that uses advanced technologies to deliver personalized cancer care based on a patient’s unique genetic and clinical profile. The use of artificial intelligence (AI) in precision oncology has shown great potential to improve diagnosis, treatment planning, and treatment outcomes. However, the integration of AI in precision oncology also raises important ethical considerations related to patient privacy, autonomy, and protection from bias. In this opinion paper, an overview is provided of previous studies that have explored the use of AI in precision oncology and the ethical considerations associated with this technology. The conclusions of these studies are compared, and the importance of approaching the use of AI in precision oncology with caution is emphasized. It is stressed that patient privacy, autonomy, and protection from bias should be made central to the development and use of AI in precision oncology. Clear guidelines and regulations must be established to ensure that AI is used ethically and for the benefit of patients. The use of AI in precision oncology has the potential to revolutionize cancer care, but it should be ensured that it striked a balance between advancements in technology and ethical considerations. In conclusion, the use of AI in precision oncology is a promising development that has the potential to improve cancer outcomes. However, ethical considerations related to patient privacy, autonomy, and protection from bias must be central to the development and use of AI in precision oncology.

Precision oncology is an exciting and rapidly evolving field that uses advanced technologies to deliver personalized cancer care based on a patient’s unique genetic and clinical profile [1]. The use of artificial intelligence (AI) in precision oncology has shown great potential to improve diagnosis, treatment planning, and treatment outcomes. However, as with any new technology, there are important ethical considerations to be addressed [2]. The development of AI in precision oncology has been driven by the exponential growth in medical data and the need to extract meaningful insights from this data. AI algorithms can analyze large datasets of medical images, genomic data, and clinical data to identify patterns and correlations that may not be visible to the human eye [3]. These patterns can inform personalized treatment decisions and improve cancer outcomes. Despite the potential benefits of AI in precision oncology, there are also significant ethical concerns. These include issues around patient privacy, autonomy, and the potential for bias in AI algorithms. In this opinion paper, these ethical issues will be discussed and several ways to balance advancements in technology with patient privacy and autonomy are explored.

The use of AI in precision oncology relies heavily on large datasets of medical information. There is a risk that this information could be accessed by unauthorized individuals or entities, leading to a breach of patient privacy. In addition, patients may be hesitant to share their personal information with healthcare providers if they believe their information may be shared without their consent [4]. It is essential that appropriate safeguards are put in place to protect patient privacy and ensure that their personal information is not misused or disclosed without their consent.

Patients have a right to participate in decisions about their treatment and to be fully informed about the risks and benefits of different options. However, the use of AI in precision oncology may lead to decisions being made about a patient’s treatment without their input or consent [5]. This can lead to a loss of control over their own healthcare and a lack of trust in the healthcare system. It is important that AI is used in a way that respects patient autonomy and promotes shared decision-making between patients and healthcare providers.

AI algorithms are only as unbiased as the data they are trained on. There is a risk that AI algorithms may perpetuate biases or inequalities in healthcare by reflecting the biases of the data they are trained on [6]. For example, if a dataset is biased towards a particular demographic group, the AI algorithm may be less accurate in predicting outcomes for individuals from other demographic groups. It is important that AI algorithms are trained on diverse datasets and regularly audited to ensure that they do not perpetuate biases or inequalities [7].

Several studies have explored the use of AI in precision oncology and the ethical considerations associated with this technology. A study by Topol [8] discussed the potential of AI to transform cancer care but also highlighted the need for safeguards to protect patient privacy and ensure that AI is used in a way that respects patient autonomy. Another study by Castaneda et al. [9] emphasized the importance of transparency and accountability in the development and use of AI in precision oncology. They called for clear guidelines and regulations to ensure that AI is used ethically and for the benefit of patients. While both studies highlighted the need for ethical considerations in the use of AI in precision oncology, they differed in their emphasis [9]. Topol [8] focused on the potential benefits of AI in precision oncology, while Castaneda et al. [9] emphasized the need for clear guidelines and regulations to ensure that AI is used ethically. However, both studies agreed that the use of AI in precision oncology must be approached with caution and that patient privacy, autonomy, and protection from bias must be central to the development and use of AI in precision oncology.

In fact, the use of AI in precision oncology is a promising development that has the potential to revolutionize cancer care. However, this technology must be approached with caution and ensure that patient privacy, autonomy, and protection from bias are central to its development and use. It is crucial that clear guidelines and regulations are established to ensure that AI is used ethically and for the benefit of patients. The use of AI in precision oncology has the potential to improve cancer outcomes, but it should be ensured that we strike a balance between advancements in technology and ethical considerations.

In conclusion, the use of AI in precision oncology shows great potential in improving patient outcomes and advancing the field of oncology. However, it is essential to approach the use of AI in precision oncology with caution and address the ethical considerations associated with this technology. There is a need to improve the education process for both oncology medical personnel and patients to ensure that they are adequately informed about the benefits and limitations of AI in precision oncology. Some key findings from various studies on AI applications in precision oncology, as well as ethical considerations related to the use of AI in these applications, were summarized in Table 1. Further research is needed to optimize the use of AI in precision oncology while ensuring patient privacy, autonomy, and protection from bias [10].

**Table 1:** Overview of studies and ethical considerations related to AI applications in precision oncology

**Study**	**AI application**	**Key findings**	**Ethical considerations**	**Reference**
Kelly et al. (2019)	Patient outcomes prediction	AI can predict patient outcomes such as hospital readmission and mortality	Need for transparency in AI models and consideration of potential biases or discrimination	[11]
Bi et al. (2019)	Patient monitoring and early detection of cancer recurrence	AI can improve the detection of cancer recurrence and enable early intervention	Need for regulation of AI in patient monitoring, protection of patient privacy and informed consent, and ethical considerations for patient autonomy and access to treatment	[12]
Dias and Torkamani (2019)	Genetic testing	AI can predict the risk of hereditary cancer based on genetic data	Need for transparency in AI models and protection of genetic data privacy	[13]
Mudgal and Das (2020)	Radiology imaging interpretation	AI outperformed radiologists in detecting cancer	Need for oversight and regulation of AI in radiology to ensure patient safety and protection from bias	[14]
Schwendicke et al. (2020)	Treatment planning and clinical decision-making	AI can improve treatment outcomes and reduce costs	Need for transparent and explainable AI models, protection of patient privacy, and consideration of ethical implications for patient autonomy	[15]
Reddy et al. (2020)	Clinical trials and drug development	AI can improve patient selection for clinical trials and accelerate drug development	Need for transparent and explainable AI models, protection of patient privacy and informed consent, and ethical considerations for equitable access to new treatments	[16]
Razzak et al. (2020)	Early cancer diagnosis	AI can detect cancer at an earlier stage than traditional methods	Need for data privacy and security to protect patient information and prevent misuse of data	[17]
Carter et al. (2020)	Risk prediction and screening	AI can improve the accuracy of breast cancer screening and risk prediction	Need for informed consent, privacy protection, and consideration of the potential harms of overdiagnosis	[18]
Huynh et al. (2020)	Tumor segmentation and radiotherapy planning	AI can improve the accuracy of tumor segmentation and radiotherapy planning	Need for clinical validation, transparency, and regulation to ensure patient safety	[19]
Hartl et al. (2021)	Development of precision medicine treatments	AI can identify novel drug targets and improve drug efficacy	Need for regulation of AI in drug development, protection of patient privacy and informed consent, and ethical considerations for drug pricing and access	[20]
Muller et al. (2021)	Personalized treatment recommendations	AI can identify effective treatment options based on genetic and clinical data	Need for informed consent and patient education to ensure understanding of AI-based recommendations	[21]
Delso et al. (2021)	Clinical trial design	AI can optimize clinical trial design and recruitment	Need for ethical considerations such as consent, privacy protection, and potential biases	[22]
Ahmad et al. (2021)	Pathology interpretation	AI can assist in pathology interpretation and reduce errors	Need for validation, transparency, and consideration of potential biases or errors	[23]
Alabi et al. (2021)	Prognosis prediction	AI can predict cancer prognosis and survival rates based on clinical and genomic data	Need for ethical guidelines and regulations for the use of AI in prognostic applications	[24]
Luk et al. (2022)	Predictive modeling for cancer diagnosis and risk stratification	AI can accurately predict cancer risk based on patient data	Need for transparent and explainable AI models, protection of patient privacy, and ethical considerations for informed consent and non-discrimination	[25]

## Abbreviations

AI: artificial intelligence

## Declarations

### Copyright

© The Author(s) 2023.
